# A Biopython-based method for comprehensively searching for eponyms in Pubmed

**DOI:** 10.1016/j.mex.2021.101264

**Published:** 2021-02-14

**Authors:** Toby C. Cornish, Larry J. Kricka, Jason Y. Park

**Affiliations:** a Department of Pathology, University of Colorado School of Medicine, Aurora, CO, USA; b Department of Pathology & Laboratory Medicine, Perelman School of Medicine at the University of Pennsylvania, Philadelphia, PA, USA; c Department of Pathology and the Eugene McDermott Center for Human Growth and Development, Children's Medical Center, and University of Texas Southwestern Medical School, Dallas, TX, USA

**Keywords:** PubMed, Medline, Eponym, Citation, Gastrointestinal diseases, Literature search, Biopython, Bibliometrics

## Abstract

Eponyms are common in medicine; however, their usage has varied between specialties and over time. A search of specific eponyms will reveal the frequency of usage within a medical specialty. While usage of eponyms can be studied by searching PubMed, manual searching can be time-consuming. As an alternative, we modified an existing Biopython method for searching PubMed. In this method, a list of disease eponyms is first manually collected in an Excel file. A Python script then creates permutations of the eponyms that might exist in the cited literature. These permutations include possessives (e.g., ‘s) as well as various forms of combining multiple surnames. PubMed is then automatically searched for this permutated library of eponyms, and duplicate citations are removed. The final output file may then be sorted and enumerated by all the data fields which exist in PubMed. This method will enable rapid searching and characterization of eponyms for any specialty of medicine. This method is agnostic to the type of terms searched and can be generally applied to the medical literature including non-eponymous terms such as gene names and chemical compounds.

• Custom Python scripts using Biopython's Bio.Entrez module automate the search for medical eponyms.
• This method can be more broadly used to search for any set of terms existing in PubMed.

## Graphical abstract

**Figure:**
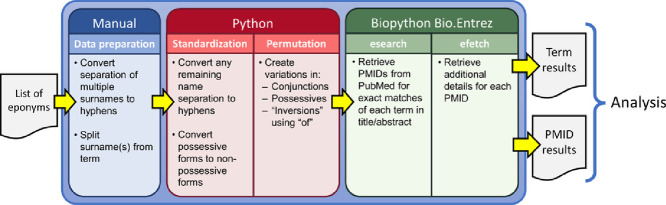

Specifications table

**Table:** 

Subject Area:	Medicine and Dentistry
More specific subject area:	*Informatics*
Method name:	*Comprehensive search of eponyms in PubMed*
Name and reference of original method:	P.J.A. Cock, T. Antao, J.T. Chang, B.A. Chapman, C.J. Cox, A. Dalke, I. Friedberg, T. Hamelryck, F. Kauff, B. Wilczynski, M.J.L. de Hoon, Biopython: freely available Python tools for computational molecular biology and bioinformatics, Bioinformatics 25 (11) (2009) 1422–1423.
Resource availability:	*If applicable, include links to resources necessary to reproduce the method (*e.g. *data, software, hardware, reagent)* Code and data: https://github.com/cornish/pubmed-eponyms Python 3: https://www.python.org/ Biopython: https://biopython.org/

## *Method details

### Method details

An “eponym” is a person after which something is named, usually due to a major role in its invention, description, or discovery. Alternatively, “eponym” may refer to the thing itself as a shorthand for an “eponymous term”. In clinical medicine, eponyms (in the latter sense) describe diseases, procedures, methods, signs, and symptoms. Eponyms enter into usage for a variety of reasons including acknowledgment or honoring an individual for a discovery. A disease eponym may succinctly communicate the etiology, histopathology, and outcome of a disorder. A typical study of eponyms is performed manually by searching the literature for the usage of a single eponym across multiple publications over a period of time. Any study of eponyms is complicated by the proliferation of variant forms over time, all of which would need to be manually generated, individually searched and then reconciled. To address inefficiencies in this process, we present a method for automating the search of a group of eponyms across the entire literature database PubMed.

## Procedure

Here we describe a method for automating PubMed searches for eponyms. This method uses custom Python scripts and modules from the Biopython package. Biopython is an open source package written in Python and C that provides bioinformatics tools in Python [1]. In addition to tools for manipulation of biological sequences and information, Biopython also has modules that can query the various NCBI databases (including PubMed) via the Entrez search engine.

### Identification and standardization of eponyms

A list of medical eponyms is required to begin the search. This list can be derived from textbooks, journal articles, or online compendia. To illustrate the method, a list of twenty-seven gastrointestinal medical eponyms (root eponyms) was manually collected from review articles [2,3]. The list of root eponyms was then standardized and saved as a comma-separated value (CSV) file. The standardization process consists of:

1. Convert compound eponyms (with more that one name) separated by a space to be separated by a hyphen (manual)
2. Split each eponym into two parts: Name(s) and Term (manual)
3. Convert and remaining compound eponyms (with more than one name) to use hyphens as name separators (Python)
4. Conversion of any eponyms with a possessive form to a non-possessive form (Python)

The process above consists of first manually “cleaning up” the list of eponyms to convert compound eponyms where the names are separated only by a space to instead use a hyphen. This manual pre-processing is needed to distinguish between an eponym named after more than one person (e.g. “Mallory Weiss”) and a multi-word surname (e.g. “Van Slyke”). Manual pre-processing is required for this step because automating this process would be exceedingly difficult and prone to error.

The second step consists of splitting each eponym in the list into two parts, the Name(s) and the Term. This is also a manual step because terms may consist of one or more words. Eponyms that use prepositional phrases as a form of possession are “inverted” (e.g. “Crypts of Lieberkuhn” to “Lieberkuhn Crypts”) as a natural consequence of this process. If the name is not readily separable from the term, the Name(s) field is left empty and the entire eponym is mapped to the Term. This situation usually arises because the eponym does not include the original proper name, but instead incorporates a modification of the original name. In our example data, “*Escherichia coli*” would fall into this category as the eponym adds the Latin suffix “*-ia*”, which is used to form genus names, to Escherich, the name of the organism's discoverer. Keeping the term intact preserves the original term while preventing the generation of permutations (see *Permutation of eponyms* below), which would not make sense in this context.

Microsoft Excel (Redmond, WA) is used for the manual pre-processing, and the data is exported as a UTF-8-encoded CSV file. For use with Python, the BOM (byte order mark) is then stripped from the Excel-generated CSV using the “Convert to UTF-8” function in Notepad++ (Don Ho, https://notepad-plus-plus.org/). The remainder of the processing is done by a Python script using the above CSV file as input. All possessive forms (i.e., those ending in “’s” and “s’”) are converted to their non-possessive equivalent. Finally, any remaining conjunctions between names (i.e., “and” and “,”) are replaced by hyphens. The fully standardized form of the eponym lists is written to a UTF-8-encoded CSV file that serves as input for the next step.

### Permutation of eponyms

To identify all occurrences of these eponyms, we then use a Python script to permute the standardized form of the eponym into an exhaustive list of variants. Where applicable, the following permutations are applied combinatorially:

1. Conjunctions: “ “, “-“, “,”, “ and “, “, and”
2. Possessives: none, all names, final name only
3. Possessives ending in “s”: “s’”, “s's”
4. Prepositional phrase (“inversion”): “Term of Name(s)”

The number of variants produced depends on the number of names (n) in the eponym, the number of names ending in “s”, and if the final name in the eponym ends in an “s”. The number of variants generated (not including the standard eponym) is 0 (where n=0), 2 to 3 (where n=1), 9 to 15 (where n=2), or 14 to 32 (where n=3). See the Python script (permute_terms.py) for additional details. The permuted terms are saved in a UTF-8-encoded CSV file that serves as input for the next step.

In our example dataset, the twenty-seven root eponyms produced a total of 116 permutations. In the examples provided, “Zenker Diverticulum” describes a type of disease structure. This is a simple eponym with a single referenced individual (“Zenker”) joined to an anatomic medical term (“Diverticulum”). In addition to the standardized version (“Zenker Diverticulum”), two additional variants were produced: “Zenker's Diverticulum” and “Diverticulum of Zenker.”

An example of a more complex eponym is “Mallory-Weiss Tear”, which references two individuals. Permutation of this term results in 13 variants in addition to the standardized version (“Mallory-Weiss Tear”). While most of these variations will return no results, the intention of our permutation method is to produce an exhaustive list of *possible* variants. The range of permutations applied was based on our experience of how eponyms vary in actual usage. The ability to generate permutations is a key advantage to using this automated method to exhaustively search a database.

### Searching PubMed using biopython

The list of permuted eponyms is then used as input to a custom Python script based on the Biopython package. Biopython's Bio.Entrez module provides a pythonic interface to the NCBI's Entrez Programming Utilities (“E-utilities” or “EUtils”), and the functions in Bio.Entrez map one-to-one to E-utilities web-based application programming interface (API). A detailed description of the Entrez E-utilities can be found here: https://www.ncbi.nlm.nih.gov/books/NBK25499/. Our script uses Biopython's Bio.Entrez.esearch and Bio.Entrez.efetch functions which correspond to the Entrez ESearch and EFetch E-utilities, respectively. Biopython does not provide a complete search implementation, but it significantly simplifies interactions with the E-utilities by handling communications, including sending requests, handling errors and retries, and parsing the returned data into Python objects. A simplified pseudocode version of our core search algorithm is shown in Algorithm 1. Important aspects of searching for Pubmed eponyms are discussed below with a focus on our implementation.

**Table:** 

While seemingly obvious, it is worth noting that a study of eponym usage in the literature hinges on identifying actual usage of the eponym itself and exclusion of related terms or synonyms. For this reason, PubMed searches must be limited to exact phrases and precise fields. For example, searching PubMed with the phrase “Zenker Diverticulum” (without quotes) returns 1316 hits, searching with the phrase “Zenker Diverticulum” (with quotes) returns 1023 hits, and searching with the phrase “Zenker Diverticulum” (with quotes) and limiting the search to the Title OR Abstract fields returns only 159 hits (search date: 11/7/2020). This not-inconsiderable discrepancy is easily explainable as a side effect of how PubMed conducts searches [4]. An unqualified (“All Fields”) search in PubMed will, in addition to matching on the exact phrase in the textual fields of the publication, also match on other fields including MeSH (Medical Subject Headings) terms. MeSH is the National Library of Medicine (NLM) controlled vocabulary for indexing articles in PubMed. “Zenker Diverticulum” exists as a MeSH term (https://meshb.nlm.nih.gov/record/ui?ui=D016672), and searching “Zenker Diverticulum"[MeSH Terms] returns 973 publications, accounting for many of these excess hits. It is not entirely clear where the remainder of the excess hits come from, but the PubMed search engine does apply additional search strategies to maximize the number of publications returned. While this can be helpful when performing general literature searches, it is not appropriate for studying eponyms. For this reason, we limit our searches to exact phrases in the Title OR Abstract fields of PubMed.

Even searches for exact phrases in Titles OR Abstracts can produce spurious results if the exact phrase is not found. In these instances, PubMed will broaden the search by (1) breaking up the quoted phrase into individual words joined by “AND” then (2) progressively dropping words from the search. This can result in very misleading results, especially when combined with our process of permutation, which frequently generates variants that produce no hits. For example, querying ESearch with “Mallory-Weiss Tear”[Title/Abstract] yields 154 hits, but the search for the permutation “Mallory-Weiss' Tear"[Title/Abstract] returns 33,071 hits. Examining the returned data for the latter query reveals that the “QueryTranslation” field is “Tear[Title/Abstract]”, indicating that PubMed has returned results for a translated query instead of the original query. Obviously, a common term like “tear” is going to produce many unrelated results. To prevent this from occurring, our method checks to see if the returned data includes a 'WarningList' with the “QuotedPhraseNotFound” warning. This flag is returned by the EUtilities API when the exact quoted phrase is not found. In these cases, the “Query Translation” does not match the original search query, and we ignore these results as indicated in the Algorithm.

Our search method, embodied by the “pubmed_search_to_csv.py” script, uses Biopython's Bio.Entrez.esearch and Bio.Entrez.efetch functions. The esearch function takes several parameters, including the database (‘pubmed’), the term (the permuted eponym), and the field (‘title/abstract’). It also takes additional parameters related to how the results should be returned. Of these, retmax sets the maximum number of results (PMIDs) to be returned by the query, and retstart sets the sequential index of the first PMID to be returned. These parameters correspond directly to parameters passed to the Eutilities API. Entrez imposes a limit of 100,000 PMIDs returned by a single query, so retmax has a maximum value of 100,000. To retrieve more than 100,000 PMIDs, our method submits multiple esearch requests while incrementing the value of retstart. The PMIDs returned by esearch are collected in a Python list, and the efetch function is then used to retrieve details for the papers represented by the PMIDs. Like esearch, efetch imposes a limit on the number of results it will return. In this case, efetch will return details for up to 10,000 PMIDs per request. To retrieve more than 10,000 PMIDs, our method submits multiple efetch requests by breaking them into sub-lists of PMIDs up to a maximum length of 10,000 (“chunks”). We request the return data as text in the MEDLINE format, then store several pieces of data for each result, including PubMed ID (“PMID”), journal title (“JT”), and date of publication (“DP). The data is then saved to two results files: “term_results.csv” is a CSV file with summary data representing one permuted term per row; “pmid_results.csv” is a CSV file containing all the hits returned by Entrez, with one PMID per row. Note that the “pmid_results.csv” file may contain duplicate PMIDs within a given root term if the same PMID was matched by more than one permutation of the term. We use an additional script to remove these duplicates, and the de-duplicated version of the “pmid_results.csv” file is used to determine the combined PMID counts for a given eponym.

## Method validation

We validated our method by querying PubMed on 11/4/2020 for 27 terms (Table 1). One data output is the raw count of the permutated eponyms. Not all eponyms were identified in the search; no citations were identified for the eponyms Carman Meniscus Sign and Heister Spiral Valves or any of their permutations. Furthermore, for other root eponyms, not all types of permutations had identified citations. This raw count does not account for PubMed citations which use multiple permutations and are in duplicate.

**Table 1:** Validation of Biopython Search by Comparison with Manual Internet-Browser based Search.

Root Term	Biopython	Manual	Difference	Percent (%)

*Escherichia coli*	273,692	273,846	154	0.06
Crohn Disease	46,894	46,997	103	0.22
Kaposi Sarcoma	13,463	13,493	30	0.22
Chagas Disease	12,088	12,097	9	0.07
Behcet Disease	8918	8940	22	0.25
Barrett Esophagus	7227	7239	12	0.17
Hirschsprung Disease	5244	5257	13	0.25
Meckel Diverticulum	3798	3806	8	0.21
Vater Ampulla	2611	2617	6	0.23
Oddi Sphincter	2387	2389	2	0.08
Zollinger-Ellison Syndrome	2238	2240	2	0.09
Peutz-Jeghers Syndrome	1857	1857	0	0.00
Whipple Disease	1805	1806	1	0.06
Zenker Diverticulum	881	883	2	0.23
Boerhaave Syndrome	702	702	0	0.00
Caroli Disease	696	699	3	0.43
Menetrier Disease	595	599	4	0.67
Wirsung Duct	442	444	2	0.45
Klatskin Tumor	349	349	0	0.00
Lieberkuhn Crypts	295	296	1	0.34
Mallory-Weiss Tear	154	154	0	0.00
Santorini Duct	132	133	1	0.75
Schatzki Ring	123	124	1	0.81
Rokitansky-Aschoff Sinuses	106	106	0	0.00
Rigler Sign	17	18	1	5.56
Total	386,714	387,091	377	0.10

For Zenker Diverticulum, the root eponym has 159 citations, and the permutated term Zenker's Diverticulum has 722 citations. For Mallory-Weiss Tear, the permutations did not additional citations to a search of the root term (n=154). Interestingly, the permutation using “of” is infrequently used for these 27 root terms. Twenty-one of the root eponyms had no citations using the “of” possessive; however, there were 6 eponyms with the “of” possessive form that had citations: Diverticulum of Meckel, Crypts of Lieberkuhn, Sphincter of Oddi, Ampulla of Vater, Duct of Wirsung, Duct of Santorini. In total, the search of the permutated eponyms resulted in 386,714 citations after the removal of duplicate citations. The most frequent citation was for Escherichia coli (n=273,692) and the least frequent was for Rigler Sign (n=17).

In addition to enumerating the total citations, other data fields within PubMed can be used to characterize the eponyms. For example, the publication year ranges from 1876 to 2021 (some publications in the 2020 search are indexed for 2021 publication). In 1876, the single eponym usage from this set is Meckel Diverticulum which was in the Journal of Anatomy and Physiology. Over time, the number of citations for these 27 terms has increased consistently, reaching 14,336 citations in 2020; the 2020 assessment is an incomplete year (January to October) but is higher than the full calendar year 2019 which had 13,119 citations.

The dynamic usage of eponyms is demonstrated with Kaposi Sarcoma which had citations dramatically increased in the 1980s and 1990s with a peak of 468 citations in 1997 followed by a general decline to 357 citations in 2019, the last complete year analyzed. In comparison, the eponym Chagas Disease had consistent growth from 5 citations in 1945 to a peak of 634 citations in 2018.

The method was validated by a manual search of PubMed using the web-based search interface (https://pubmed.ncbi.nlm.nih.gov/). All permuted terms were searched and the number of search hits was recorded for each exact phrase. To ensure that the exact term was matched, the search string was enclosed in quotation marks, and the search was limited to the Title and Abstract fields using PubMed's search syntax (“[Title/Abstract]”). For example, here are the search strings used for the “Zenker Diverticulum” permutations: “Zenker Diverticulum”[Title/Abstract]; “Zenker's Diverticulum”[Title/Abstract]; “Diverticulum of Zenker”[Title/Abstract].

“Carman Meniscus Sign” and “Heister Spiral Valves” are not listed because both had zero citations by both Biopython and manual search of Pubmed.

Biopython and manual searching had similar numbers of citations identified with a difference per root term ranging from 0.0 to 5.56%. For all root terms, manual searching identified additional citations. Overall, the difference in citations identified was 0.1%.

## Conclusion

Studying eponym usage in the literature presents a unique set of challenges that differs from the usual goal of literature searches. As such, one must be careful and precise in how a tool like PubMed is used to obtain search results. We have presented a method for identifying eponym usage that automates the most tedious aspects of exhaustive searching for eponymous terms while addressing most of the pitfalls one is expected to encounter. It should be noted that while this method is designed to identify eponym usage in the textual data in PubMed (i.e. title and abstract), it cannot identify eponym usage elsewhere in the body of a paper. Currently, identifying eponyms in the full text of articles remains a tedious manual process that is highly dependent on the availability of adequate full text search tools provided by the journal itself. Given that there are approximately 30,000 journals cited in PubMed (https://www.nlm.nih.gov/bsd/serfile_addedinfo.html) an exhaustive full text search of journals for eponyms would be nigh unachievable. Additionally, access to the full text of many journal articles is restricted based on subscriptions. The methods, as presented here, can be used to select from PubMed a subset of key journals for additional manual exploration at the full text level. However, with minor modifications, this method may be applicable for full text searching of databases that include the full text of open access articles (e.g., PubMed Central) to further enhance the technique.

## Declaration of Competing Interests

None.
